# Supplementary material for: Discovery and pharmacophoric characterization of chemokine network inhibitors using phage-display, saturation mutagenesis and computational modelling
Source: Nat Commun. 2023 Sep 16;14:5763. doi: 10.1038/s41467-023-41488-z (PMC10505172; doi:10.1038/s41467-023-41488-z)
Supplement: Supplementary file 4 — Source Data [file 41467_2023_41488_MOESM4_ESM.zip › SourceData/scripts/xattune_20230807170606.tar!/xattune/analysis/attune_ci/SV_metaanalysis_plot.pdf]

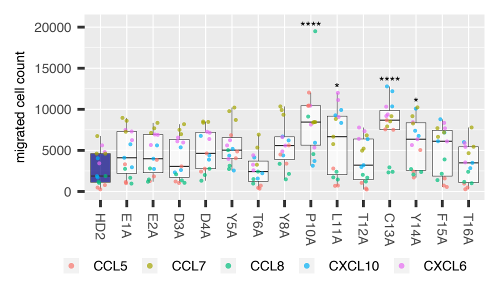

migrated cell count
20000
15000
10000
5000
0
****
*
****
*
HD2
E1A
E2A
D3A
D4A
Y5A
T6A
Y8A
P10A
L11A
T12A
C13A
Y14A
F15A
T16A
CCL5
CCL7
CCL8
CXCL10
CXCL6
